# Evolution of Microstructure and Mechanical Properties of Ultra-High-Strength Heat-Resistant Bearing Steel During Long-Term Aging at 500 °C

**DOI:** 10.3390/ma18030639

**Published:** 2025-01-31

**Authors:** Chuncheng Guo, Hongxiao Chi, Jian Zhou, Jinbo Gu, Dangshen Ma, Lili Dong

**Affiliations:** 1 Special Steel Department of Central Iron and Steel Research Institute (CISRI), Beijing 100081, China; guochuncheng0715@163.com (C.G.); zhou-jian-168@163.com (J.Z.); gujinboustb@163.com (J.G.); m2762@163.com (D.M.); dlili1120@163.com (L.D.); 2 School of Materials Science and Engineering, Kunming University of Science and Technology, Kunming 650093, China

**Keywords:** ultra-high-strength heat-resistant bearing steel, long-term aging, microstructural evolution, mechanical properties

## Abstract

In this study, various testing methods, including X-ray diffraction (XRD), scanning electron microscopy (SEM), Electron Backscatter Diffraction (EBSD), and high-resolution transmission electron microscopy (HRTEM), were utilized to examine the effects of aging time on the microstructure and mechanical properties of ultra-high-strength heat-resistant bearing steel. The findings revealed that as the aging time progressed, the tensile strength, yield strength, and elongation exhibited an initial increase followed by a decline. Specifically, after 50 h of aging, the tensile strength and yield strength peaked at 2133 MPa and 1874 MPa, respectively. Calculations indicated that precipitation strengthening was the primary contributor to the strength, accounting for 1311 MPa. During the aging process, the martensite laths underwent coarsening, broadening from 202 nm to 306.5 nm, while the residual austenite remained relatively stable. Additionally, dislocations underwent annihilation, resulting in a decrease in dislocation density to 4.84 × 10^11^/cm^2^ at 100 h. As the aging time continued to increase, both M_6_C and M_2_C phases gradually coarsened. Notably, the number of M_2_C phases increased, and they transformed from an acicular shape to a spherical shape at 100 h.

## 1. Introduction

Over the past few decades, the aircraft manufacturing industry has flourished, with a strong demand for high-speed jet engines, gas turbines, and other components driving the pursuit of high thrust-to-weight ratios (in the range of 15–20). Increasing the rotational speed of the main shaft in aerospace engines is currently an effective technical approach to enhancing thrust-to-weight ratios. Bearings are vital components in aircraft propulsion and rotor systems, and they are evolving in the direction of being able to withstand higher temperatures, higher rotational speeds, and greater loads. Therefore, aircraft bearing materials must possess high strength, high surface hardness, and high wear resistance, along with good ductility and toughness. Additionally, resistance to high-temperature oxidation and corrosion is another essential characteristic of aircraft bearings [1,2,3,4,5,6].

However, as the operating temperatures of bearing materials increase, the tempering temperatures of ultra-high-strength heat-resistant bearing steels have approached these operating temperatures, which accelerates their microstructural evolution, degrades their mechanical properties, and ultimately affects the reliability and service life of the bearings. Thus, assessing mechanical property degradation and associated microstructural evolution at operating temperatures is particularly crucial for flight safety. Researchers often study the evolution of the microstructure and mechanical properties of ultra-high-strength heat-resistant bearing steels by substituting tempering temperatures for operating temperatures to address related issues. For instance, Chen et al. [7] reported that CSS-42 L steel undergoes changes in its microstructure and mechanical properties when the tempering temperature is altered. They also concluded that M_6_C is responsible for precipitation hardening when the steel is tempered at 490 °C and 520 °C, whereas M_2_C, produced by the decomposition of residual austenite, contributes to higher strength when the tempering temperature rises to 550 °C. Zhang et al. [8] reported on the evolution of carbides in Ferrium S53 steel during tempering, transitioning from M_7_C_3_ at 400 °C to the coexistence of M_7_C_3_ and M_2_C at 482 °C, and finally to the coexistence of M_2_C and M_23_C_6_ at 630 °C. Zeng et al. [9] pointed out that for low-carbon martensitic stainless bearing steel (with a carbon content lower than CSS-42 L steel), the intrinsic strengthening mechanism shifts from dislocation–precipitation dual strengthening after tempering at 470 °C to precipitation-dominated strengthening after tempering above 530 °C as the tempering temperature increases from 470 °C to 590 °C. Chen et al. [6] noted that as tempering time increases, the width of martensite blocks increases, the austenite content rises, and the dislocation density decreases. They also observed that the number of M_2_C and M_6_C carbides first increases and then decreases, but their size continues to grow. Coarsened M_23_C_6_ carbides precipitate at grain boundaries after 336 h of tempering, and their quantity increases with further increases in tempering time. These studies primarily focus on the effects of tempering temperature, neglecting the influence of aging time on the microstructure and mechanical properties of ultra-high-strength heat-resistant bearing steels, which is often more critical for the performance of aviation bearings. Moreover, with prolonged aging time, the degradation of the mechanical properties of bearings can provide experimental data for bearing life predictions.

Therefore, we investigate ultra-high-strength heat-resistant bearing steels subjected to different aging times and discuss the relationships between aging time, microstructure, and mechanical properties.

## 2. Materials and Methods

**Figure 1:**
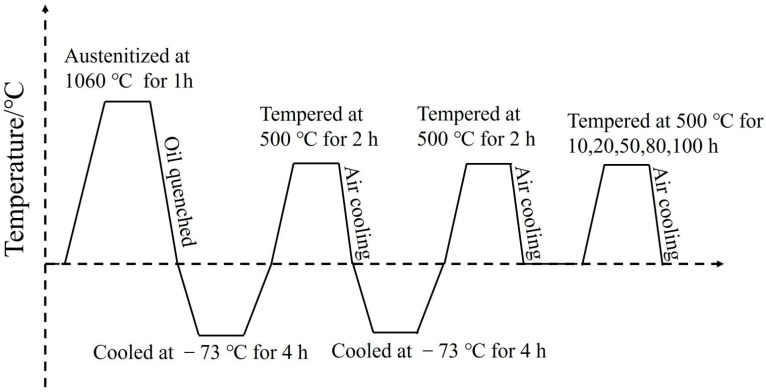
Heat treatment process curve.

The test material is ultra-high-strength heat-resistant bearing steel, with its main composition detailed in Table 1. The steel ingot was obtained through dual vacuum melting processes of Vacuum Induction Melting (VIM) and Vacuum Arc Remelting (VAR) followed by homogenization treatment. A 50 kg cast was held at 1200 °C for 1 h and then hot-forged into round bars with a diameter of 15 mm. To ensure good performance prior to use, the test steel samples were austenitized at 1060 °C for 1 h and oil-quenched. Afterward, they underwent a 4-h cryogenic treatment, followed by tempering at 540 °C and air cooling. This cryogenic treatment and tempering process was then repeated. After air cooling, the samples were subjected to aging treatments at 500 °C for durations of 10, 20, 50, 80, and 100 h. The heat treatment process diagram is illustrated in Figure 1.

Samples were cut from the heat-treated steel bars along the axial direction for tensile testing and microstructural observation. To observe the microstructure, after grinding and polishing, the samples were wiped with a solution of CuCl_2_ + 3.5 g FeCl_3_ + 2.5 mL HNO_3_ + 50 mL ethanol + 50 mL H_2_O for approximately 10 s, rinsed, and blow-dried. Subsequently, The microstructure after tempering was characterized using a scanning electron microscope (FEI Quanta 650) manufactured by FEI Company in the United States, Hillsboro, OR, USA. The ground and polished samples were electrolyzed in a chromic acid solution until their surfaces turned gray. Subsequently, the residual austenite was measured using a D8 ADVANCE X-ray diffractometer produced by Bruker AXS GmbH, Karlsruhe, Germany, under the following diffraction conditions: Co target, tube voltage of 35 kV, tube current of 40 mA, and scanning speed of 2°/min. The volume fraction of austenite in the steel was calculated using Equation (1) [8]. The dislocation density was calculated using a convolutional multiple whole profile (CMWP) program.

(1) $$V_{r}=\frac{1}{1+\frac{R_{\gamma }I_{\alpha }}{R_{\alpha }I_{R}}}$$

where *V_r_* is the volume fraction of austenite and *I_α_* and *I_γ_* are the integrated intensities of the diffraction peaks in the BCC and FCC phases, respectively. *R_α_* and *R_γ_* depend on HKL, θ, and the type of substance.

The grain orientation distribution, grain size, grain boundaries, and misorientation angle distribution of the material were characterized using Electron Backscatter Diffraction (EBSD) technology produced by EDAX, Pleasanton, CA, USA. The maximum step size for EBSD analysis was set at 0.1 micrometer with an acceleration voltage of 20 kV. The samples for measurement were prepared by conductive hot mounting followed by argon ion polishing. The EBSD data were subsequently processed using Channel 5 software. In this study, observations were made using a Tecnia G2 F20 transmission electron microscope (TEM) produced by FEI Company in the United States. To prepare the samples, metallic slices were cut from the tensile fracture and then ground down to a thickness of 0.1 mm using abrasive papers. Electrolytic double-jet polishing was conducted using a double-jet apparatus with a working current of approximately 75 mA and a working temperature of around −20 °C. The double-jet solution was a 6% hypochlorous acid alcohol solution, and the pump flow rate was adjusted as needed during the jetting process. The double-jet polished samples were observed under the Tecnia G2 F20 TEM to examine the morphology of martensitic laths, the morphology of the second phase along with its diffraction patterns, and the morphology and distribution of carbides. The width of the martensitic laths was measured in at least five TEM images using Image-Pro Plus 6.0 software.

**Figure 2:**
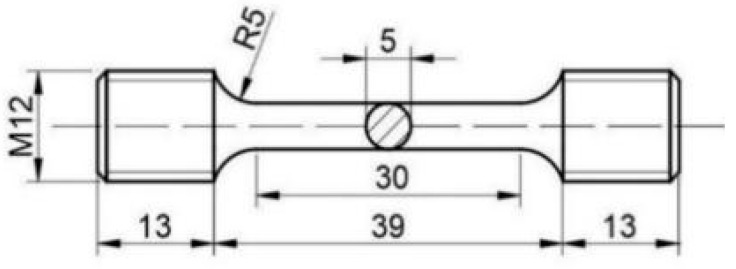
Dimensions for room temperature tensile testing.

The room-temperature tensile specimens were processed in accordance with the national standard GB/T 228-2020 [10], “Methods for tensile testing of metallic materials at ambient temperature”, with specimen dimensions of M12 × 65 mm, as shown in Figure 2 for technical drawings. The tensile tests were conducted on a universal tensile testing machine to measure the tensile strength, yield strength, percentage elongation after fracture, and reduction in area of the tested steel. The final values obtained were the averages of three parallel specimens.

## 3. Results

### 3.1. Microstructural Evolution

**Figure 3:**
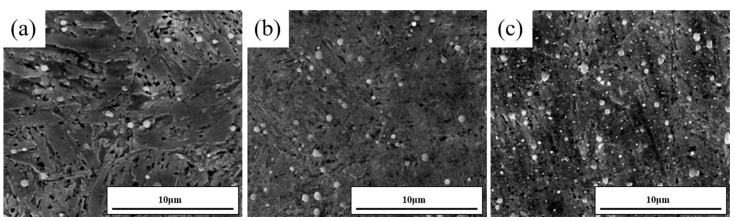
SEM images illustrate the aging microstructure of the experimental steel after being held at 500 °C for durations of (**a**) 0 h, (**b**) 50 h, (**c**) 100 h.

Figure 3a,c display the microstructure of the tested steel at different aging times. As shown in Figure 3a, at the 0 h state, small amounts of carbides are evenly dispersed within the martensitic matrix. As the tempering time increases to 50 h, the size of the large carbides remains almost unchanged, but their number increases. The martensite boundaries undergo slight recovery, and the precipitation of a small number of fine-sized carbides can be observed. After reaching 100 h of aging, the number of large carbides remains almost constant, but they undergo coarsening, while a large number of fine-sized carbides precipitate and grow within the martensite matrix.

**Figure 4:**
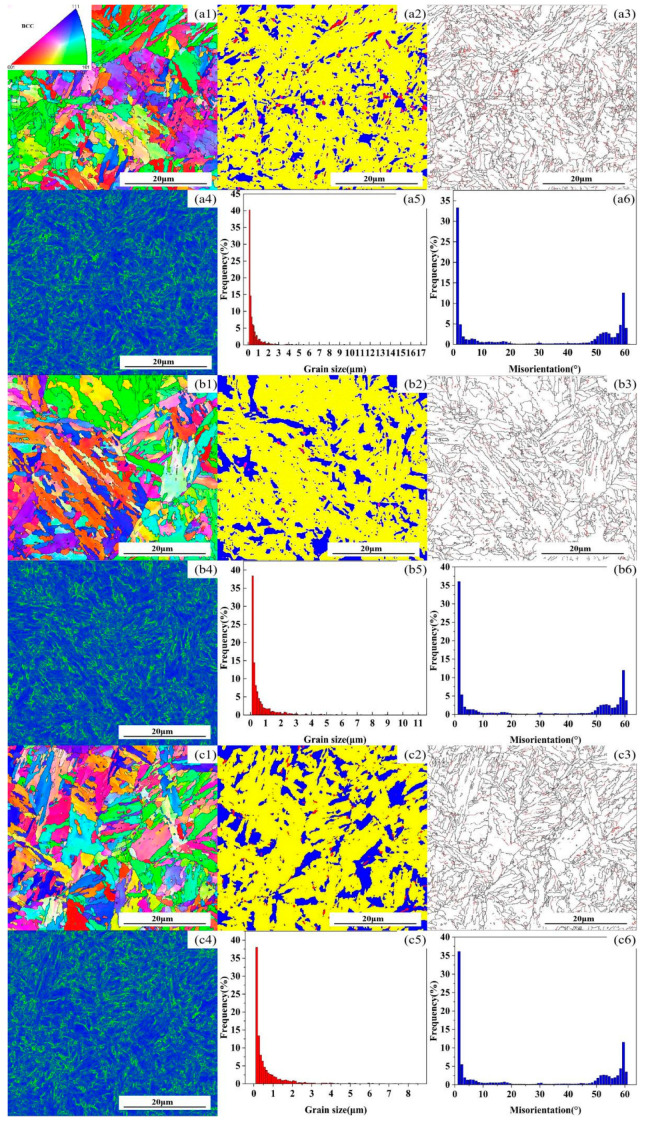
IPF coloring orientation images depicted with grain boundaries for conditions of (**a**) 0 h, (**b**) 50 h, and (**c**) 100 h: (**a1**,**b1**,**c1**) and (**a2**,**b2**,**c2**) represent recrystallization maps, (**a3**,**b3**,**c3**) represent maps of large-angle and small-angle grain boundaries, (**a4**,**b4**,**c4**) represent Kernel Average Misorientation (KAM) maps, (**a5**,**b5**,**c5**) represent grain size distribution maps, and (**a6**,**b6**,**c6**) represent grain boundary misorientation frequency maps.

**Figure 5:**
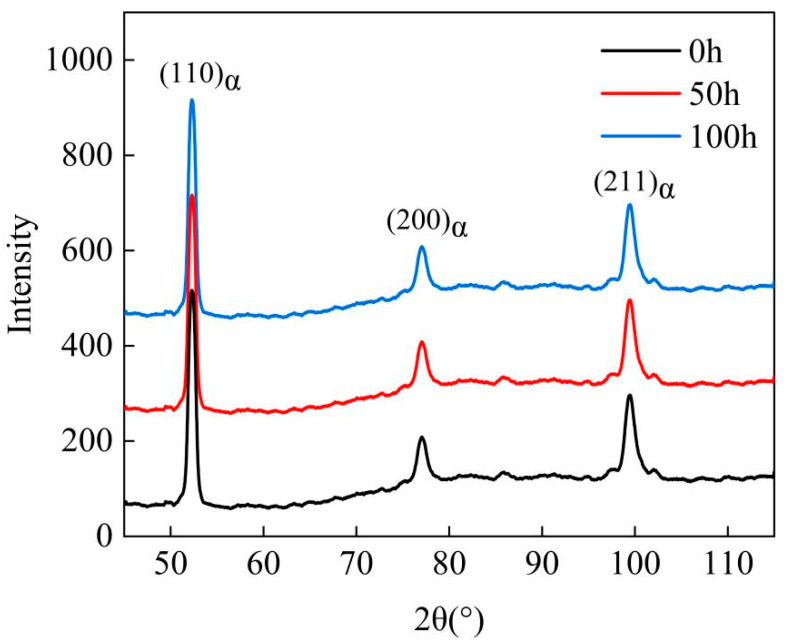
XRD of experimental steel at different aging times.

With the extension of the aging time, in addition to the precipitation and growth of carbides, changes in the martensite substructure and orientation relationship also occur [11,12,13,14,15,16,17,18]. The inverse pole figure (IPF) color-coded orientation maps of the microstructure of the tested steel after aging for 0, 50, and 100 h are shown in Figure 4(a1,b1,c1). It can be seen from the figures that some small martensitic substructures with HAGBs disappear, while some larger ones with HAGBs become coarser as the holding time increases. The phase maps are presented in Figure 4(a2,b2,c2), where yellow represents the recovered portion of the tested steel, blue indicates the recrystallized portion, and red denotes the deformed grains. The figures reflect that as the aging time increases, the number of deformed grains decreases, and the recrystallization phenomenon becomes more pronounced. The misorientation distribution maps are shown in Figure 4(a3,b3,c3), where red represents low-angle grain boundaries (LAGBs, with misorientation angles between 2° and 15°), and black represents high-angle grain boundaries (HAGBs, with misorientation angles greater than 15°). LAGBs are formed by the rearrangement of dislocations, so they are actually dislocation accumulation zones. When the number of LAGBs decreases, it means that the distribution of dislocations in the crystal becomes sparser, i.e., the dislocation density decreases. The Kernel Average Misorientation (KAM) maps are shown in Figure 4(a4,b4,c4), mainly representing the accumulation of dislocations. They show the same situation as Figure 4(a4,b4,c4) and are consistent with the XRD test results, indicating that the dislocation density decreases as aging time increases. Histograms of the grain size distribution are shown in Figure 4(a5,b5,c5). Calculations reveal that the average grain size increases with aging time, being 2.09, 2.39, and 2.45 μm, respectively. Histograms of the grain misorientation distribution are shown in Figure 4(a6,b6,c6), indicating that the grain misorientation does not significantly increase with the increase in holding time. The X-ray diffraction patterns of the tested steel after different holding times are shown in Figure 5. Table 2 provides the specific values of retained austenite and dislocation density, showing that the volume fraction of retained austenite is negligible, while the dislocation density gradually decreases.

**Figure 6:**
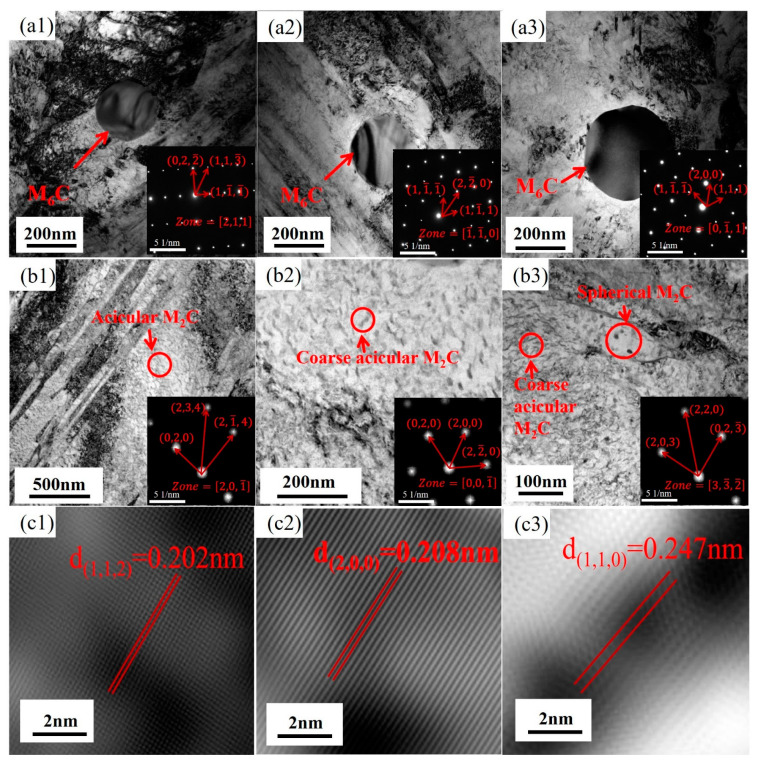
High-resolution transmission electron microscope (HRTEM) morphologies of carbides at different aging times: (**a1**) M_6_C 0 h, (**a2**) M_6_C 50 h, and (**a3**) M_6_C 100 h; (**b1**) M_2_C0 h, (**b2**) M_2_C 50 h, and (**b3**) M_2_C 100 h; the inverse Fourier transform (IFT) images are: (**c1**) 0 h M_2_C, (**c2**) 50 h M_2_C, and (**c3**) 100 h M_2_C.

**Figure 7:**
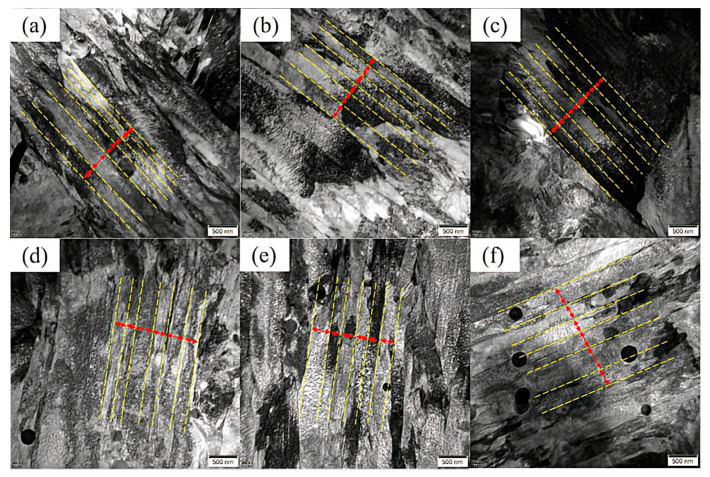
High-resolution transmission electron microscope (HRTEM) images showing martensitic laths in the experimental steel after aging for (**a**) 0 h, (**b**) 10 h, (**c**) 20 h, (**d**) 50 h, (**e**) 80 h, and (**f**) 100 h.

Figure 6 illustrates the distribution and morphology of carbides at various aging times. In Figure 6(a1–a3), it can be observed that as the aging time extends from 0 h to 100 h, the spherical carbides enlarge. Their radius is approximately 160.5 nm at 0 h, increases to 172 nm at 50 h, and undergoes a significant change at 100 h, reaching about 235 nm. Through diffraction pattern indexing, these carbides are identified as the M_6_C phase. As shown in Figure 6(b1–b3), in addition to the large M_6_C phase, there are numerous fine precipitates in the tested steel. Figure 6(b1) displays a large number of needle-like carbides dispersed within the structure at 0 h aging time. Based on high-resolution transmission electron microscope (HRTEM) analysis, the selected area marked in red is for electron diffraction, and after extension indexing, it is confirmed to be the M_2_C phase. Figure 6(b2) presents the microstructure after 50 h of aging, when the needle-like carbides have coarsened into short, rod-like shapes. The selected area marked in red undergoes electron diffraction, and upon extension indexing, it is identified as the M_2_C phase. Figure 6(b3) shows the microstructure after 100 h of aging, when the carbides are present as both short, rod-like and spherical shapes. The short, rod-like carbides are coarsened M_2_C phase, and the spherical carbides, upon diffraction indexing, are also confirmed to be the M_2_C phase. Figure 6(c1–c3) measure the interplanar spacings of the needle-like M_2_C; short, rod-like M_2_C; and spherical M_2_C. The interplanar spacing for needle-like M_2_C is d(1,1,2) = 0.202 nm; for short, rod-like M_2_C is d(1,1,0) = 0.247 nm; and for spherical M_2_C is d(2,0,0) = 0.208 nm [17,18]. During the long-term aging process, the width of the martensite laths also undergoes changes. As depicted in Figure 7a–f, the martensite lath width is 202 nm at 0 h aging time (Figure 7a), increases to 247 nm at 50 h (Figure 7d), and further increases to 306.5 nm at 100 h (Figure 7f). The results indicate that the martensite laths coarsen with the extension of the aging time [19].

### 3.2. Tensile Properties

**Figure 8:**
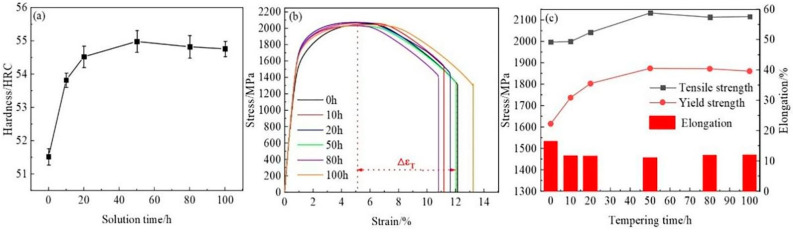
Changes in mechanical properties of the experimental steel after different aging times: (**a**) hardness variation curve, (**b**) stress–strain curve, and (**c**) diagrams showing changes in strength and elongation.

Figure 8 illustrates the mechanical properties of the tested steel at different holding times. As the holding time increases, the hardness of the tested steel gradually rises, reaching a peak value of 55HRC at 50 h, as shown in Figure 8a. Figure 8b,c present the stress–strain curves and corresponding tensile properties of the tested steel after various holding times. It can be observed that at 0 h, the tensile strength and yield strength of the tested steel are 1997 MPa and 1615 MPa, respectively. As the holding time extends, both the tensile strength and yield strength of the tested steel increase after being held for 10 h and 20 h. After 50 h, the tensile strength and yield strength of the tested steel reach their maximum values, which are 2133 MPa and 1874 MPa, respectively. Subsequently, as the holding time continues to increase, the tensile strength and yield strength of the tested steel decline slightly, reaching 2116 MPa and 1861 MPa, respectively, at 100 h.

**Figure 9:**
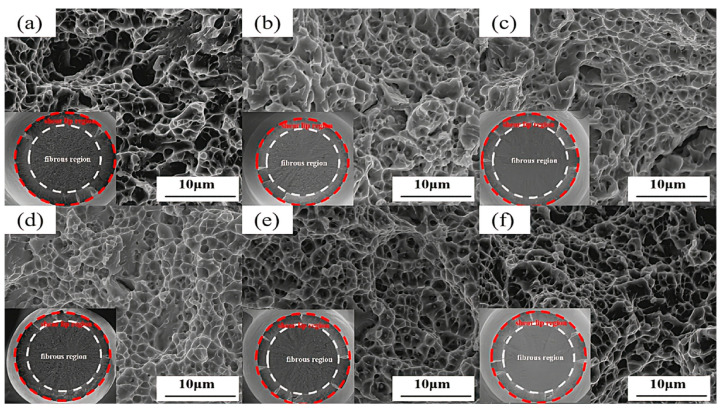
The scanning electron microscope (SEM) images showing tensile fracture surfaces of the experimental steel after aging for (**a**) 0 h, (**b**) 10 h, (**c**) 20 h, (**d**) 50 h, (**e**) 80 h, and (**f**) 100 h.

Figure 9a–f display the tensile fracture morphologies of the tested steel at different aging times. The microscopic fracture morphology images are taken from the fibrous zone of each individual sample, and the macroscopic fracture morphologies of the tensile samples are shown in the lower left corner of each image. It can be observed from Figure 9a–f that the fracture planes of the tensile samples are all composed of a central fibrous zone and a shear lip zone, with their average radii varying with the tempering time. The fracture surfaces of the samples are elliptical in shape, with r_f_ and r_F_ representing the average radius of the fibrous zone and the average fracture radius, respectively. These were measured four times and averaged, with the specific values presented in Table 3. The elongation of the tested steel first decreases and then increases with the extension of aging time, reaching a minimum at 50 h.

## 4. Discussion

### 4.1. The Influence of Aging Time on Microstructural Evolution

The influence of aging time on the microstructure is primarily manifested in the aspect of carbides. Large carbides are mainly M_6_C, as shown in Figure 6(a1–a3). With the extension of aging time, M_6_C gradually grows larger, mainly through diffusion and interface reactions. The finer carbides are primarily M_2_C. As aging time increases, the number of M_2_C carbides increases, and they undergo coarsening, which can be explained by the Ostwald ripening mechanism. Specifically, C atoms and alloy elements dissolved in the austenite during quenching are inherited into the martensitic matrix. During subsequent tempering, these soluble C atoms and alloy elements form carbides within the martensitic matrix or at substructure boundaries. When aging reaches 100 h, the M_2_C phase transforms into a spherical shape, which is closely related to the diffusion behavior of C atoms. This diffusion not only occurs within the carbides but also involves diffusion between the carbides and the matrix. As aging time increases, the diffusion process of the atoms becomes more intense, leading to changes in carbide morphology. The increase in the number and coarsening of carbides results in a reduction in the C content within the martensitic matrix.

**Figure 10:**
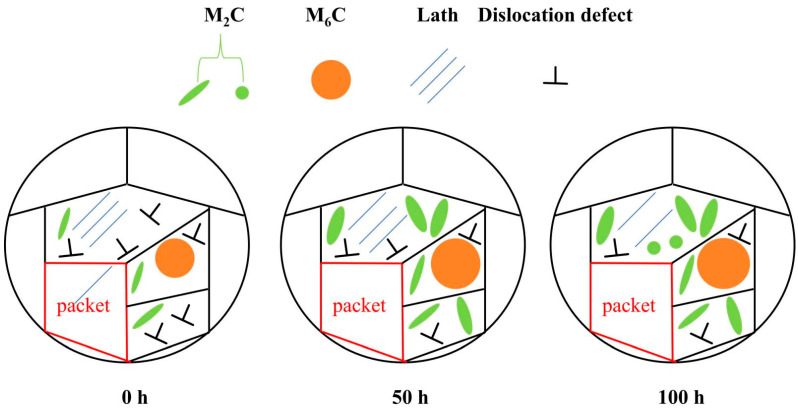
Schematic diagram illustrating the microstructural evolution of the experimental steel after aging for different durations.

In addition to its impact on carbides, aging time also affects the martensitic substructure. As shown in Figure 7a–f, the width of the martensitic laths broadens. Furthermore, compared with bulk martensitic laths, the small misorientation angles between the martensitic laths are more prone to merging [20,21,22,23,24,25,26]. The residual austenite content in the tested steel is extremely low and can be neglected. The influence of aging time on the microstructural evolution of the tested steel is illustrated in Figure 10.

### 4.2. The Influence of Aging Time on Strength

As mentioned previously, the increase in tempering time leads to changes in the microstructure, which subsequently cause variations in strength. When discussing the relationship between microstructure and strength, the strength enhancements arising from solid solution strengthening, grain boundaries, dislocations, and precipitation should be considered. Since the volume fraction of retained austenite in this steel is relatively low, its influence on strength is not taken into account. The lath boundaries can be regarded as high-angle boundaries, and the strength increment can be calculated according to the Hall–Petch formula. The strength of this steel can be calculated by considering the following aspects [26,27,28,29,30]:

(2) $$\sigma =\sigma_{0}+\sigma_{ss}+k_{HP}d^{-1/2}+M\alpha Gb\rho_{0}^{1/2}+\sigma_{ppt}$$

Here, *σ*_0_ represents the internal fictitious stress of body-centered cubic (BCC) iron, which is 50 MPa; *σ_ss_* denotes the strength contribution from solid solution strengthening; *k_HP_* is the Hall–Petch slope, which is 120 MPa μm^1/2^; d is the average spacing of the martensite laths; M is the Taylor factor, which is 2.8 for BCC; α is equal to 0.2 times the dislocation density; G is the shear modulus, which is 80.7 GPa; b is the Burgers vector, which is 0.248 nm; *ρ*_0_ represents the initial dislocation density; and *σ_ppt_* is the strength increment due to the precipitation of nanoparticles.

The C content in the test steel is low, and the C atoms, at 3 × 10^3^, can be dissolved into the dislocations, so that all the C atoms in the quenching process are dissolved into the dislocations of the slat martensite. So that the solid solution strengthening increment does not take into account the C atoms, the change in the strength of *σ_ss_* can be calculated according to the empirical Formula (3) [31,32,33]:

(3) $$\sigma_{ss}=11[\%Mo]+31[\%Cr]+3[\%V]+30[\%Ni]+1067[\%W]+368[\%Co]$$

The strength increment from the second-phase particles is contributed by either dislocation bypassing or shearing of coherent particles, depending on the type, structure, and size of the precipitation [34,35,36,37]. There are two kinds of particles in the current alloy: M_6_C carbides and M_2_C carbides. The strength increment caused by M_6_C is dominated by the bypassing mechanism, which can be expressed via the Ashby–Orowan Equation (4):

(4) $$\sigma_{M_{6}C}=(0.26\frac{Gb}{r_{M_{6}C}})f^{\frac{1}{2}}ln(\frac{r_{M_{6}C}}{b})$$

*rM*_6_*C* and *f* represent the average radius and volume fraction of M_6_C particles, respectively. When the average radius of M_6_C particles *rM*_6_*C* is 160.5 nm, 172 nm, and 235 nm, and their volume fractions f are 0.049%, 0.062%, and 0.071%, respectively, the calculated strength increments *σM*_6_*C* caused by M_6_C particles are approximately 4.6 MPa, 4.9 MPa, and 40.6 MPa, respectively.

*σ_M_*_2*C*_ can be based on the theory of Gladman et al. using the Ashby–Orowan modified model (the use of the original formula), and the reinforcement of precipitates can be expressed by the following equation [34,35,36,37]:

(5) $$\sigma_{M_{2}C}=\frac{10\mu b}{5.72\pi^{3/2}r}f^{1/2}\ln\left(\frac{r}{b}\right)$$

where *r* is the atomic radius (μm); *μ* is the shear coefficient, which is 80.26 × 10^3^ MPa for steel; *b* is the Burr’s vector, which takes the value of 2.48 × 10^−4^ μm; and *f* is the volume fraction of precipitated particles (%) [11]:

(6) $$f=\left(\frac{1.4\pi }{6}\right)\left(\frac{{Nd}^{2}}{A}\right)$$

where *f* and *d* represent the volume fraction and average radius of the carbides, respectively, while *N* denotes the number of carbides in a region, and *A* is the area of the selected region. The volume fraction and average size of nano-sized precipitates in the TEM images were statistically analyzed using Image Pro-Plus software. Five micro-areas were selected for the statistics, and the average values were taken, as shown in Table 4.

**Figure 11:**
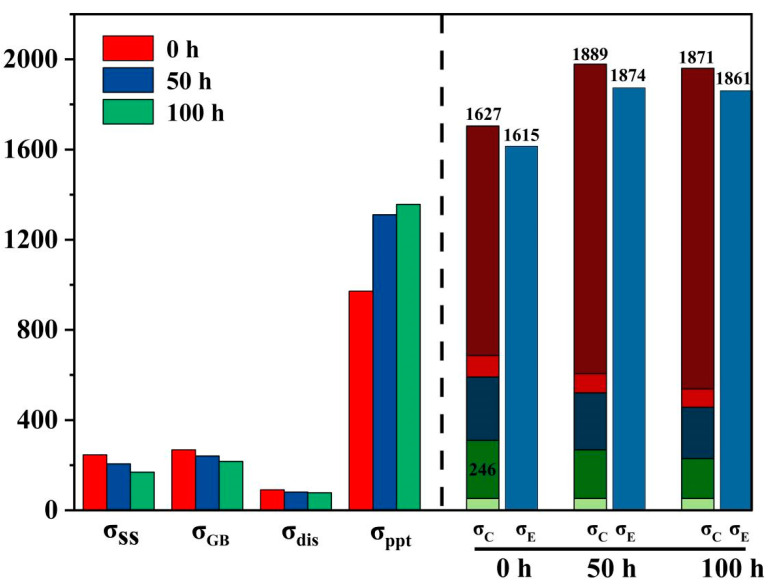
Calculated strength contributions from each mechanism: solid solution strengthening (σ_SS_), grain boundary strengthening (σ_GB_), dislocation strengthening (σ_dis_), precipitation strengthening (σ_ppt_), calculated yield strengthening (σ_C_), and the experimental date (σ_E_).

Figure 11 summarizes the strengthening contributions of each individual mechanism. It is noteworthy that the strengthening contributions from precipitation hardening are approximately 972 MPa, 1311 MPa, and 1357 MPa, respectively, while the total strengthening increments calculated from other mechanisms are approximately 655 MPa, 531 MPa, and 514 MPa, respectively. This indicates that precipitation hardening is the primary factor contributing to the overall strengthening effect, The detailed data are shown in Table 5.

### 4.3. The Influence of Aging Time on Ductility

As shown in Figure 8c, the elongation of the tested steel decreases as the aging time increases, reaching a minimum at 50 h, followed by a slight upward trend. The variation in elongation is primarily related to the precipitation and growth of precipitates, the softening degree of the matrix structure, and the accumulation of dislocations. According to Figure 3a–c, when the aging time is short, the precipitates are fine in size and coherent with the austenitic matrix. However, as the aging time prolongs, the precipitates transition from a coherent relationship with the austenitic matrix to a semi-coherent or non-coherent relationship, and the precipitates undergo coarsening. At this point, the precipitates, which initially increased the dislocation density, evolve into inclusions within the austenitic matrix. When subjected to external forces, they are prone to becoming crack initiation points, leading to easier fracture of the steel during tensile testing and reduced elongation. As shown in Figure 9c,d, obvious secondary cracks appear on the fracture surface of the tested steel. The softening of the matrix structure results in decreased elongation. Conversely, as aging progresses, the hardness of the tested steel’s matrix exhibits a trend of first increasing and then slightly decreasing (as shown in Figure 8a). As indicated in Table 2, as aging progresses, the dislocation density decreases, and the accumulation of dislocations weakens, which can improve the elongation of the tested steel to a certain extent.

Differences in the plasticity of the tested steel are also evident on the tensile fracture surfaces. The tensile fracture surfaces all exhibit fiber zones and shear lip zones. Large, deep dimples appear on the tensile fracture surface at 0 h. As the tempering time increases, the dimples become shallower and smaller. Secondary cracks appear on the tensile fracture surfaces of the tested steel at 20 h and 50 h. However, at 80 h and 100 h, the secondary cracks disappear, and quasi-cleavage fracture characteristics emerge on the tensile fracture surfaces.

In martensitic steels, reversed austenite has a profound impact on plasticity. Some believe that although reversed austenite can enhance elongation through the transformation-induced plasticity (TRIP) effect resulting from strain-induced martensitic transformation, the TRIP effect becomes significant only when its content reaches a relatively high level. In this work, the volume fraction of reversed austenite is low, and the TRIP effect can be neglected [37,38,39,40].

Mao et al. reported on the relationship between inhomogeneous plastic deformation and the crack propagation rate when the tensile fracture surface consists solely of fiber zones and shear lip zones, as indicated by Equation (7) [11].

(7) $$\nu =\frac{\left[r_{f}\times \varepsilon /\left(R_{0}-r_{F}\right)\right]}{{\Delta \varepsilon }_{T}}$$

where ν and Δε_T_ represent the average crack propagation rate and the amount of inhomogeneous plastic deformation, respectively; ε is the strain rate, which is 1 mm/min in this experiment. The original radius of the tensile specimen is 2500 μm, and r_f_ and r_F_ denote the average radius of the fiber zone and the average fracture radius of the shear lip, respectively, with their values shown in Table 4. Based on the equation, the average crack propagation rate is calculated, and the results are also presented in Table 4. The crack propagation rate is opposite to the trend of elongation, meaning that the higher the elongation, the slower the crack propagation rate.

## 5. Conclusions

(1) As the aging time prolongs, the size of the M_6_C phase increases, and the number and size of the M_2_C phase also increase, changing from needle-like to spherical shapes. The width of the martensite laths increases, the content of residual austenite is negligible, and the dislocation density decreases.

(2) The tensile strength and yield strength of the tested steel reach their peak values at 50 h, which are 2133 MPa and 1874 MPa, respectively. Precipitation strengthening dominates the contribution to the strength. As aging progresses, several other strengthening mechanisms gradually weaken, while the precipitation strengthening mechanism gradually enhances.

(3) As the aging time increases, the elongation of the tested steel decreases, reaching its lowest value at 50 h, followed by a slight upward trend. At 0 h, large, deep dimples appear on the tensile fracture surface. As the tempering time increases, the dimples become shallower and smaller. Secondary cracks appear on the fracture surfaces at 20 h and 50 h, while at 80 h and 100 h, the secondary cracks disappear, and quasi-cleavage fracture characteristics emerge on the tensile fracture surfaces.

## Figures and Tables

**Table 1:** Chemical composition of test steels (mass fraction, %).

Elemental	C	Cr	Ni	Mo	Co	W	V	Nb
Content	0.18	10	4	5	12.68	0.53	0.62	0.029

**Table 2:** Volume fraction of residual austenite and dislocation density in steels tested with different tempering times.

Time (h)	Volume Fraction of Austenite (vol. %)	Dislocation Density (10^11^/cm^2^)
0 h	0.54	6.63
50 h	0.55	5.15
100 h	0.56	4.84

**Table 3:** Mean radius of steel fiber zone and shear lip for different tempering time tests (r_F_: the average fracture radius; r_f_: the average radius of the fibrous zone).

Time (h)	r_F_ (μm)	r_f_ (μm)	Crack Propagation Rate (×10^−3^ μm/s)
0 h	1658	1079	34.3
10 h	1629	1286	46.8
20 h	1714	1314	48.9
50 h	1595	1243	37.8
80 h	1697	1303	54.1
100 h	1714	1257	42.8

**Table 4:** Volume fraction (%) and average radius (nm) of the M_2_C phase at different holding times.

Time (h)	f (%)	r (nm)
0 h	0.24	12.5
50 h	1.05	24.1
100 h	1.12	35.6

**Table 5:** Incremental contribution of different intensities.

Time/h	σ_0_/MPa	σ_ss_/MPa	σ_GB_/MPa	σ_dis_/MPa	σ_ppt_/MPa	σ/MPa
0 h	50	246	268	91	972	1627
50 h	50	206	241	81	1311	1889
100 h	50	169	217	78	1357	1871

## Data Availability

The original contributions presented in this study are included in the article. Further inquiries can be directed to the corresponding author.

## References

[1] Tomasello C.M., Burrrier H.I., Knepper R.A., Balliett S., Maloney J.L. (2002). Progress in the evaluation of CSS-42L™: A high performance bearing alloy. Am. Soc. Mech. Eng..

[2] Klecka M.A., Subhash G., Arakere N.K. (2013). Microstructure–Property Relationships in M50-NiL and P675 Case-Hardened Bearing Steels. Tribol. Trans..

[3] Luo H., Wang X., Liu Z., Yang Z. (2020). Influence of refined hierarchical martensitic microstructures on yield strength and impact toughness of ultra-high strength stainless steel. J. Mater. Sci. Technol..

[4] Perez M., Sidoroff C., Vincent A., Esnouf C. (2009). Microstructural evolution of martensitic 100Cr6 bearing steel during tempering: From thermoelectric power measurements to the prediction of dimensional changes. Acta Mater..

[5] Luo Z.-J., Shen J.-C., Su H., Ding Y.-H., Yang C.-F., Zhu X. (2010). Effect of Substructure on Toughness of Lath Martensite/Bainite Mixed Structure in Low-Carbon Steels. J. Iron Steel Res. Int..

[6] Chen X., Zheng L., Feng S., Li J., Wang F., Zhang H. (2022). Tempering influence on microstructural evolution and mechanical properties in a core of CSS-42L bearing steel. Mater. Sci. Eng. A.

[7] Chen H., Zeng T., Shi Q., Wang N., Zhang S., Yang K., Yan W., Wang W. (2023). Microstructure evolution and mechanical properties during long-term tempering of a low carbon martensitic stainless bearing steel. J. Mater. Res. Technol..

[8] Zhang W.W. (2022). Study on the Precipitation Behavior of Laves Phase and Its Effect on Thermal Deformation and Mechanical Properties of Thermo-Span Alloys.

[9] Zeng T., Li W., Wang N., Wang W., Yang K. (2022). Microstructural evolution during tempering and intrinsic strengthening mechanisms in a low carbon martensitic stainless bearing steel. Mater. Sci. Eng. A.

[10] (2022). Metallic Materials—Tensile Testing—Part 1: Method of Test at Room Temperature. GB/T 228.1-2020.

[11] Ma H.X., Li Y.G. (2002). Measurement of size distribution and volume fraction of precipitates in silicon steel. Mater. Sci. Eng. A.

[12] Mao C., Liu C., Yu L., Li H., Liu Y. (2018). Mechanical properties and tensile deformation behavior of a reduced activated ferritic-martensitic (RAFM) steel at elevated temperatures. Mater. Sci. Eng. A.

[13] Zhang H., Ponge D., Raabe D. (2014). Designing quadplex (four-phase) microstructures in an ultrahigh carbon steel. Mater. Sci. Eng. A.

[14] Zhang B., Zhao M., Dong Y., Misra R., Du Y., Wu H., Du L. (2021). On the structure-property relationship in a novel 1000 MPa hot-rolled TRIP steel with strain-assisted ferrite transformation. Mater. Sci. Eng. A.

[15] Chen S.-C., Wang Y.-T., Lin Y.-C., Huang C.-Y., Yang J.-R., Yen H.-W. (2019). Microstructure and mechanical behaviors of GPa-grade TRIP steels enabled by hot-rolling processes. Mater. Sci. Eng. A.

[16] Wei W., Ke J., Liu Z., Liu G., Liu Q., Qian D., Hua L. (2023). Understanding the microstructural evolution and fretting wear behaviors of M50 bearing steel heat treated at different temperatures. J. Mater. Res. Technol..

[17] He X., Yang L.X., Wu Z.W., Zhao K.Y., Yang M.S. (2019). Effects of cryogenic treatment on M6C carbide in 16Cr14Co12Mo5Ni2 bearing steel. Trans. Mater. Heat Treat..

[18] Du N., Liu H., Cao Y., Fu P., Sun C., Liu H., Li D. (2021). Formation mechanism of MC and M2C primary carbides in as-cast M50 bearing steel. Mater. Charact..

[19] Li S., Zhu G., Kang Y. (2016). Effect of substructure on mechanical properties and fracture behavior of lath martensite in 0.1C–1.1Si–1.7Mn steel. J. Alloys Compd..

[20] Ma X., Wang L., Liu C., Subramanian S. (2012). Microstructure and properties of 13Cr5Ni1Mo0.025Nb0.09V0.06N super martensitic stainless steel. Mater. Sci. Eng. A.

[21] Li Y., Yan W., Cotton J.D., Ryan G.J., Shen Y., Wang W., Shan Y., Yang K. (2015). A new 1.9GPa maraging stainless steel strengthened by multiple precipitating species. Mater. Des..

[22] Zhou P., Yang W., Wu Y., Zong Y. (2023). Characterization of microstructural evolution with pre-strain in BG801 bearing steel: Grain, carbides, retained austenite and martensite. Vacuum.

[23] Liu W., Cao Y., Guo Y., Xu B., Sun M., Li D. (2020). Characteristics and transformation of primary carbides during austenitization in Cr4Mo4V bearing steel. Mater. Charact..

[24] Zhao Z., Li C., Li Z., Liu T., Zhi M., Zhu J., Sun J. (2003). Study on the strengthening phase in a ultra-high strength stainless gear steel. Aeronaut. Mater..

[25] Li S., Zhao K., Wang K., Yang M. (2017). Microstructural evolution and thermal stability after aging of a cobalt-containing martensitic bearing steel. Mater. Charact..

[26] Zhang Y., Zhan D., Qi X., Jiang Z. (2019). Effect of tempering temperature on the microstructure and properties of ultrahigh-strength stainless steel. J. Mater. Sci. Technol..

[27] Li S., Xiao M., Ye G., Zhao K., Yang M. (2018). Effects of deep cryogenic treatment on microstructural evolution and alloy phases precipitation of a new low carbon martensitic stainless bearing steel during aging. Mater. Sci. Eng. A.

[28] Semba H., Abe F. (2013). Alloy design and creep strength of advanced 9%Cr USC boiler steels containing high concentration of boron. Energy Mater..

[29] Ribárik G., Ungár T., Gubicza J. (2010). MWP-fit: A program for multiple whole-profile fitting of diffraction peak profiles by ab initio theoretical functions. J. Appl. Crystallogr..

[30] Liu T., Cao Z., Wang H., Wu G., Jin J., Cao W. (2020). A new 2.4 GPa extra-high strength steel with good ductility and high toughness designed by synergistic strengthening of nano-particles and high-density dislocations. Scr. Mater..

[31] Gu J., Li J., Chang R., Li L. (2019). Comprehensive effect of nitrogen on Cr-Mo-V hot-working die steel with enhanced strength and toughness. Mater. Sci. Eng. A.

[32] Shi R., Wang Z., Qiao L., Pang X. (2019). Microstructure evolution of in-situ nanoparticles and its comprehensive effect on high strength steel. J. Mater. Sci. Technol..

[33] Niu M.C., Zhou G., Wang W., Shahzad M.B., Shan Y., Yang K. (2019). Precipitate evolution and strengthening behavior during aging process in a 2.5 GPa grade maraging steel. Acta Mater..

[34] Ning A.G. (2015). Research on the Integrated Strengthening Mechanism of Nanoscale Precipitates and Steel in Hot Work Die Steel.

[35] Yu D.G., Tan Y.B. (1983). Organizational Strength of Steel.

[36] Gladman T. (1992). Grain Refinement in Multiple Microalloyed Steels. HSLA Steels: Processing, Properties and Application.

[37] Wang J.-S., Mulholland M.D., Olson G., Seidman D. (2013). Prediction of the yield strength of a secondary-hardening steel. Acta Mater..

[38] Zhou L., Tang G., Ma X., Wang L., Zhang X. (2018). Relationship between microstructure and mechanical properties of M50 ultra-high strength steel via quenching-partitioning-tempering process. Mater. Charact..

[39] Su Y., Wang J., Yu X., Wang S., Xia Y., Liu L., Liu J. (2021). Effect of deep tempering on microstructure and hardness of carburized M50NiL steel. J. Mater. Res. Technol..

[40] Won Y.-J., Kwon Y.-J., You J.-S., Park S.-S., Cho K.-S. (2023). Role of W addition in reducing heat checking and enhancing the mechanical properties of hot work tool steel. J. Mater. Res. Technol..

